# Eccentric exercise versus Usual-care with older cancer survivors: The impact on muscle and mobility- an exploratory pilot study

**DOI:** 10.1186/1471-2318-11-5

**Published:** 2011-01-27

**Authors:** Paul C LaStayo, Robin L Marcus, Lee E Dibble, Sheldon B Smith, Susan L Beck

**Affiliations:** 1 Department of Physical Therapy, University of Utah, Salt Lake City, Utah, USA; 2 College of Nursing, University of Utah, Salt Lake City, Utah, USA

## Abstract

**Background:**

Resistance exercise programs with high compliance are needed to counter impaired muscle and mobility in older cancer survivors. To date outcomes have focused on older prostate cancer survivors, though more heterogeneous groups of older survivors are in-need. The purpose of this exploratory pilot study is to examine whether resistance exercise via negative eccentrically-induced work (RENEW) improves muscle and mobility in a diverse sample of older cancer survivors.

**Methods:**

A total of 40 individuals (25 female, 15 male) with a mean age of 74 (± 6) years who have survived (8.4 ± 8 years) since their cancer diagnosis (breast, prostate, colorectal and lymphoma) were assigned to a RENEW group or a non-exercise Usual-care group. RENEW was performed for 12 weeks and measures of muscle size, strength, power and mobility were made pre and post training.

**Results:**

RENEW induced increases in quadriceps lean tissue average cross sectional area (Pre: 43.2 ± 10.8 cm^2^; Post: 44.9 ± 10.9 cm^2^), knee extension peak strength (Pre: 248.3 ± 10.8 N; Post: 275.4 ± 10.9 N), leg extension muscle power (Pre: 198.2 ± 74.7 W; Post 255.5 ± 87.3 W), six minute walk distance (Pre: 417.2 ± 127.1 m; Post 466.9 ± 125.1 m) and a decrease on the time to safely descend stairs (Pre: 6.8 ± 4.5 s; Post 5.4 ± 2.5 s). A significant (P < 0.05) group x time interaction was noted for the muscle size and mobility improvements.

**Conclusions:**

This exploration of RENEW in a heterogeneous cohort of older cancer survivors demonstrates increases in muscle size, strength and power along with improved mobility. The efficacy of a high-force, low perceived exertion exercise suggests RENEW may be suited to older individuals who are survivors of cancer.

**Trial Registration:**

ClinicalTrials.gov Identifier: NCT00335491

## Background

Maintaining an at-risk older individual's mobility and function is a top priority in aging research [1]. Older cancer survivors represent an exemplar and needy target group because cancer and its treatment are associated with accelerated functional decline [2]; due, in part, to low physical activity levels [3] and deficits in muscle and mobility [4,5].

Only 1/5^th ^of older cancer survivors engage in exercise consistent with public health recommendations despite reports that exercise can improve fatigue levels [6], quality of life [7], and strength [8], all predictors of continuing to exercise at recommended levels [9]. Moreover, the older cancer survivor's admitted muscle weakness limits their functional mobility (e.g., prolonged walking and stair climbing) [3-5]. For this reason, resistance exercise is the optimal mode of exercise to counter impaired mobility and the muscular side effects of cancer such as muscle wasting and weakness. Additionally, a high-intensity resistance exercise program is ideal as the positive effects on muscle strength, cardiopulmonary function, quality of life, and fatigue effects can be maintained for at least one year [10].

A recent systematic review [8] of resistance exercise in cancer survivors identified only two (out of 24) studies dedicated solely to resistance exercise in older (65 years of age or older) survivors. Segal [11] implemented a supervised program (3 times per week for 12 weeks) with older (mean age = 68 years) survivors and demonstrated improvements in muscle strength in men on androgen deprivation therapy following prostate cancer versus a control group. In a subsequent study, Segal [12] noted 24 weeks of resistance training in an identical group improved quality of life, levels of fatigue, aerobic fitness and strength. While accumulating evidence supports the efficacy of resistance training, many older cancer survivors who avoid or are unable to perform resistance exercise will experience an accelerated functional decline as muscle and mobility deficits constitute the most influential risk factors for accidental falls in older populations with chronic conditions [13]. Unfortunately, the older cancer survivor has numerous barriers to exercise that ultimately interfere with improved function [6], hence exercise modes with high participation rates that focus on muscle strengthening and improved mobility are a high priority.

A high-intensity resistance exercise via negative, eccentrically-induced work (RENEW) program is safe and feasible for older individuals, including prostate survivors [14], and can promote positive clinical changes in muscle [15] and mobility [16,17]. Its efficacy, however, has not yet been demonstrated in an older diverse cohort of cancer survivors. The novelty of RENEW as a potential physical exercise countermeasure for older cancer survivors is that only low levels of exertion are required to produce relatively high (~2-4-fold greater) muscle workloads and commensurate positive changes in muscle and mobility [15,18]. This benefit may be especially important for older individuals who have limited energy to put towards exercise due to the combination of aging and long-term functional decline associated with having survived cancer and its treatment.

We therefore conducted an exploratory pilot study to examine whether RENEW improves muscle and mobility in a diverse sample of older cancer survivors. We hypothesized that RENEW would induce improvements following the intervention period while those following a course of usual-care would not demonstrate improvements.

## Methods

The study was approved by the Institutional Review Board at the University of Utah and was in accordance with the Declaration of Helsinki. All participants provided written informed consent prior to participation. We used a restricted randomization design to ensure a balanced allocation into the arms of the study. This exploratory pilot study took place in an academic health science center setting. Participants were allocated to a RENEW intervention or a usual-care control group (Usual-Care) after using randomized blocks of varying length (2 and 4), to approximate balance between treatment arms. Sealed group assignment envelopes were opened after screening and consent were completed. Because this was an exploratory pilot study and it was important to have adequate numbers in each group, after a dropout the next group assignment was to that respective group as a replacement. Outcome measures were assessed by study personnel blinded to group assignment at baseline and at the end of the intervention period (12 weeks).

### Recruitment and Eligibility

We employed a two-part approach to recruitment from 2006-2008 which included receiving names and identifying information from: 1) clinical databases at the University of Utah and Huntsman Cancer Institute, and 2) individuals responding to announcements in a Salt Lake City daily newspaper, or via word of mouth referral. This exploratory pilot study was designed to recruit a sample of 40 cancer survivors, 20 participants randomized to each group (RENEW and Usual-Care). The a priori sample size calculation needed to detect a treatment effect, defined as the difference in change scores in the muscle and mobility variables between the RENEW and usual care groups, with 90% power and an alpha level of 0.05 was 18 participants per group. The clinical database at the Huntsman Cancer Institute and the data warehouse at the University of Utah hospitals and clinics resulted in a pool of over 3000 patients from which we identified 286 cancer survivors who met the eligibility criteria related to age, diagnosis, and disease status. Each of these potential participants received a personal letter providing information about the study and a prompt to return a postcard to the study coordinator denoting no further contact be made regarding potential participation; 19% exercised this opt-out option. The 93 individuals responding to announcements were contacted directly via phone to assess their interest and screen for eligibility. All community ambulating males and females 60 years of age or older surviving cancer (breast, prostate, colorectal, lung or lymphoma), with no evidence of disease, and at least 6 months post-treatment (local = surgery and/or radiation; or systemic = chemotherapy; or a combination of local and systemic), and a Folstein Mini-Mental State Examination score > 23 were eligible. Participants were included if they had moderate levels (≥4 on a scale from 0-10) of fatigue and/or weakness as measured by the General Fatigue Scale and General Weakness Scale respectively. Those who met inclusion and were enrolled in the study also provided a list of comorbidities and their level of health related quality of life via a structured interview process and completion of the Short-Form 36 questionnaire respectively. Individuals were excluded if they had a central nervous system disorder (e.g., multiple sclerosis, Parkinson's disease), neurological insult (e.g., cerebrovascular accident), chronic fatigue syndrome, or myopathic or rheumatological disease that adversely impacts skeletal muscle structure/function or manifests in a mobility disorder. Engaging in regular (2-3 times per week) exercise (aerobic or resistance) during the preceding 6 months constituted another reason for exclusion.

### RENEW Intervention

**Figure 1:**
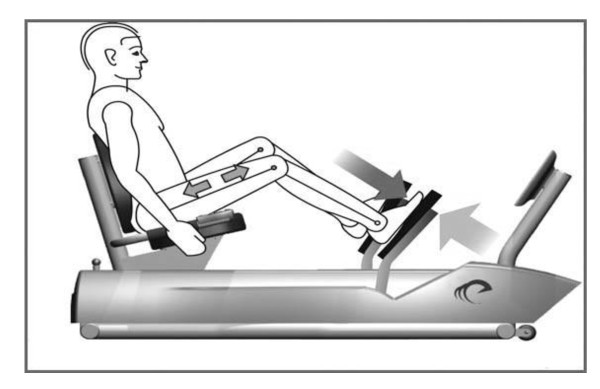
**High muscle forces are generated on an eccentric stepper (Eccentron; BTE Technologies, Inc., Hanover, MD, USA) powered by a 2-horsepower motor that drives the pedals**. As the pedals move toward the participant (largest arrow), the rider resists by applying force to the pedals (arrow at foot level). Because the magnitude of force produced by the motor exceeds that produced by the rider, the leg extensors (arrows in thigh) work eccentrically (lengthening), creating negative work.

The lower extremity RENEW exercise occurred on a recumbent eccentric stepper with a focus on the knee extensor (quadriceps) muscle group as previous studies [15-17] have noted clinically beneficial changes in the quadriceps with RENEW and positive relationships between quadriceps function and mobility in older individuals [16]. An exercise specialist in a health science center-clinical setting supervised each participant's exercise session. Prior to training, the stepper seat setting was individually adjusted to each participant's leg length, and safety guidelines were reviewed. The recumbent eccentric stepper was powered by a 2-hp motor that drives the foot pedals in a "backward" direction (i.e., toward the individual). Eccentric muscle contractions occurred when the participant attempted to resist this motion by pushing on the pedals (with verbal instruction to "try to slow down the pedals") as the pedals moved toward the participant. Because the magnitude of the force produced by the stepper exceeded that of the participant's, the pedals continued to move toward the participant at a constant velocity (15-20 cycles per minute), resulting in eccentric contractions of the knee and hip extensors, including the quadriceps muscles (Figure 1).

The subjects began with thrice weekly, 3-5 minute sessions on the stepper for the first 2 weeks and progressed to a maximum of 15 minutes over the next 3 to 4 weeks and attempts were made to perform RENEW on the stepper for 16-20 minutes for the last 8 weeks. The progression of the eccentric exercise work rate was determined as a function of the perceived exertion (RPE) using a "target" workload on a computer monitor and is summarized in Table 1. Once the subjects achieved an RPE of "somewhat hard," they were instructed to maintain that RPE for the duration of the exercise program.

**Table 1:** Perceived Exertion and RENEW Progression: The frequency and duration of RENEW and the rating of perceived exertion over the 12 Week Training Period

Week	Times/Week	Training Duration	Rating of Perceived Exertion
1	3	3-5 minutes	7 (very, very light)
2	3	5 minutes	9 (very light)
3	3	6-10 minutes	11 (fairly light)
4	3	11-15 minutes	11-13 (fairly light to somewhat hard)
5-12	3	16-20 minutes	11-13 (fairly light to somewhat hard)

### Usual-Care

The Usual-Care group did not participate in the RENEW exercise program, but continued with their oncology follow-up care. Since the participants were cancer survivors this generally included a recommendation to call their oncologist or primary health care provider if any symptoms became problematic. Follow-up oncology appointments were at 6-month intervals and moved to annual visits after a year or two of continued remission.

### Measures

#### Muscle Size: Quadriceps Lean Tissue

Muscle size (cm^2^), i.e., the average mid-thigh cross-sectional area (CSA) of lean skeletal muscle tissue of the quadriceps was determined using magnetic resonance image (MRI) of both thighs. Participants were placed supine in a 1.5 Tesla whole body MR imager (Signa Lightening LX 8.4; General Electric Medical Systems, Milwaukee, WI). To establish the region of interest (ROI), a coronal fast spoiled gradient echo scout scan was used to identify the superior and inferior boundaries of the scans (the femoral head and the tibiofemoral joint line). Once the ROI was established, axial T1 weighted images were acquired in the standard body coil using a fast-spin echo sequence with repetition time/time to echo = 550/9.2, 8-mm slice thickness, 15-mm interslice distance, and a 320 X 320 matrix. Four images from the middle 1/3 of each thigh were used to determine average CSA of lean tissue using custom written image analysis software (MatLab; Mathworks, Natick, MA). Manual tracing eliminated subcutaneous fat and bone and isolated the fascial border of the thigh to create a subfascial ROI. The total number of pixels within the ROI, a frequency distribution and a histogram of all pixels and signal intensities produced a specific peak designated as lean tissue. The same investigator, blinded to time point of the scan and slice location, performed measurements of individual participants before and after training. This technique has demonstrated high levels of intrarater reliability with an average interclass correlation coefficient of 0.99 and the validity of the measurement was determined by analysis of images obtained from a cadaveric thigh phantom [16].

#### Muscle Strength: Maximal Voluntary Isometric Knee Extension Peak Force

Knee extension peak strength (N) was quantitatively assessed by unilateral maximal voluntary isometric efforts on a KinCom dynamometer (Chattanooga Inc., Hixon, TN.). Both lower extremities were tested and these strength measures were assessed prior to and two-five days following the training interventions. Participants were seated and their knees were fixed at 90° of flexion and they were stabilized by chest and thigh straps. Participants were asked to fold their arms across their chest while performing these tests. Prior to testing, participants practiced submaximal contractions at 50% and 75% of their maximal effort. Three practice trials were then performed. After a brief rest period, three separate maximal contractions were performed, each held for 5 seconds with a 3-minute rest between trials. The outcome variable knee extension peak strength was calculated as the average of both lower extremity peak force trials. The test of isometric knee extension peak force with an isokinetic dynamometer machine has excellent reliability with ICCs > 0.99 [19].

#### Muscle Power: Stair Climbing Leg Power

Leg extension muscle power (W) was assessed with a simple, clinically-utilized timed stair climb power test [20]. Stair climb leg muscle power was calculated with the following formula: power equals force times velocity. Stair climb time (s) and vertical height of the stairs (m) were used to calculate velocity (distance/time), and body mass (kg) and acceleration (m/s^2^) due to gravity were used to calculate force. At the base of a well-lighted, 10-stair flight participants were instructed to safely ascend the stairs as fast as they could when the examiner said, "Ready, set, go." Timing began once the participant began moving. When both feet of a participant reached the top step, the timing stopped. Time was recorded to the nearest 0.01 s, and the average of 3 trials was taken. The stair climb power measure is clinically relevant as it is associated with more complex modes of testing leg power impairments in older individuals (20) and the test-retest reliability is excellent with ICCs > 0.94 [21].

#### Mobility: Six-Minute Walk and Stair Descent Tests

The six-minute walk test, an assessment of mobility used to assess overall locomotion and endurance, measures the distance (m) walked in six minutes. Participants were asked to cover as much distance as possible within six minutes without running. The six-minute walk test has high test-retest reliability in older populations with various co-morbid conditions [22,23].

The stair descent test of mobility relies almost exclusively on eccentric muscle contractions. Since mobility tasks requiring graded eccentric contractions are compromised more in old individuals than is the ability to perform concentric contractions while ascending stairs, impaired eccentric control may contribute to the greater number of falls during stair descent. Therefore, we employed the stair descent test of mobility by asking participants to descend one flight of stairs under close or contact supervision as quickly and safely as possible. Time was recorded to the nearest 0.01 second from a verbal go signal to final foot placement on a standard flight of 10 stairs and the average of three trials was recorded. Previous research has supported the high reliability of this measure of mobility with ICCs > 0.97 [24].

### Statistical Analyses

Data were analyzed with Sigma Stat Version 3.5 (Chicago, IL). Descriptive statistics were calculated for demographic variables and dependent measures. The assumptions of parametric statistical tests were assessed via tests of normality and homogeneity of variance. In all cases, the assumptions were met and therefore parametric tests were performed. In the analyses, we evaluated the effect of RENEW and Usual Care on muscle size, strength, power and mobility. First, pre-intervention baseline values for the dependent variables for RENEW and the Usual-Care groups were compared and tested with independent sample t-tests. Next, in order to control for potential pre-intervention differences between the groups, the primary aim was examined with a 2-way repeated-measures analysis of covariance (ANCOVA), with group (RENEW versus Usual-Care) as the between-subjects variable and time (Pre and Post intervention) as the within subject variable for each dependent variable: muscle size, strength, power and mobility. In all cases, the pre-intervention value for each dependent variable was used as the covariate. Because our hypotheses were focused on the differential response of the groups, our primary interest was the interaction between the group x time effects. In order to fully understand what changes drove any observed interaction effects or time main effects, and to examine and compare the magnitude of within group changes, interval estimators of pre to post intervention differences (95% confidence intervals of the mean differences) as well as calculation of within group effect sizes (ES) were utilized. The level of statistical significance for all tests for differences was set at p < 0.05.

## Results

### Participants

The participants included a total of 40 individuals (25 female, 15 male) with a mean age of 74 (± 6) years and a body mass index of 28 (± 6) (table 2). The cohorts mean survival was 8.4 (± 8) years since their cancer diagnosis. Additionally, a description of all participants' baseline levels of fatigue and weakness, and their health related quality of life, are depicted in table 2.

**Table 2:** Characteristics of the 40 participants randomized into the RENEW (females n = 13, males n = 7) and Usual-Care (females n = 12, males n = 8) groups: The mean and standard deviation (SD) of: age, survival in years, body mass index, self-reported levels of fatigue and weakness from the General Fatigue Scale and General Weakness Scale respectively, and the physical and mental health-related quality of life (HRQOL) from the Short Form-36

	RENEW	Usual-Care
Age in Years	75 (7)	73 (5)
Survival in Years	8.5 (9)	8.3 (7)
Body Mass Index	28 (4)	29 (7)
Self Report of Fatigue on 0-10 scale	4.8 (1.7)	4.9 (2.1)
Self Report of Weakness on 0-10 scale	4.2 (1.7)	4.4 (2.1)
Physical-HRQOL	42 (9)	39 (10)
Mental-HRQOL	56 (4)	51 (15)

The prevalence of cancer types, comorbid disease conditions and cancer treatments amongst the cohorts is depicted in table 3; breast = 55%, prostate = 28%, colorectal = 18%, lung = 5%, and lymphoma = 3%. Arthritis and hypertension were the most frequently listed comorbidities amongst the participants. The participants' previous treatment for their cancer(s) was characterized as either a local treatment (surgery ± radiation therapy) = 70%, a systemic treatment (chemotherapy) = 10%, or a combination of a local and systemic treatment = 20%.

**Table 3:** The absolute number and relative (%) prevalence of cancer types, comorbid disease conditions and cancer treatments amongst the cohorts

	RENEW	Usual-Care
Cancer Type		
Breast	10 (50%)	12 (60%)
Prostate	4 (20%)	7 (35%)
Colorectal	4 (20%)	3 (15%)
Lymphoma	1 (5%)	0 (0%)
Lung	1 (5%)	2 (10%)
Comorbidities		
Arthritis	14 (29%)	12 (29%)
Hypertension	13 (27%)	10 (24%)
Heart Disease	5 (10%)	6 (15%)
Lung Disease	5 (10%)	5 (12%)
Gastric Ulcers	3 (6%)	4 (10%)
Diabetes	4 (8%)	2 (5%)
Stroke or TIA	2 (4%)	1 (2%)
Kidney Disease	2 (4%)	1 (2%)
Neuromuscular	1 (2%)	0 (0%)
Cancer Treatment		
Local	14 (70%)	13 (65%)
Systemic	2 (10%)	2 (10%)
Local and Systemic	4 (20%)	5 (25%)

**Figure 2:**
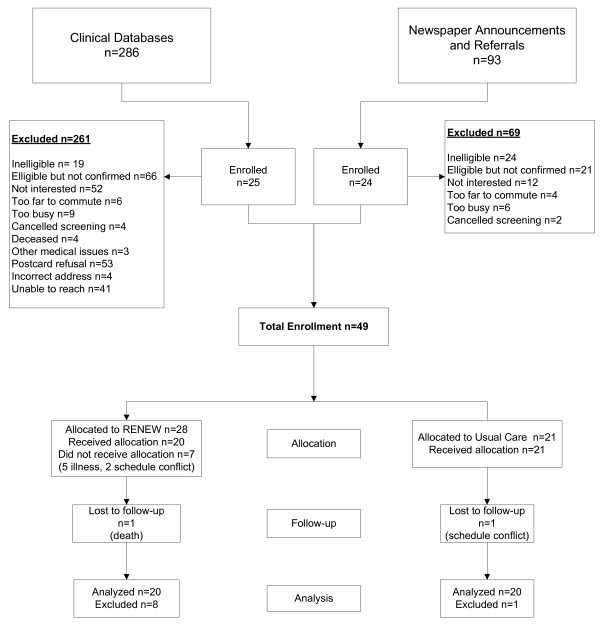
**The consolidated standards of reporting trials (CONSORT) diagram for the study**.

The restricted randomization design ensured age and sex balance between treatment groups and resulted in 20 individuals per group being analyzed. See the Consolidated Standards of Reporting Trials (CONSORT) diagram (Figure 2). Following the restricted randomization, 28 individuals were assigned to the RENEW group and 21 individuals to the Usual-Care group. Following the dropout of individuals during the allotted intervention period (n = 7 from the RENEW group) or post-testing period (n = 1 from the RENEW group) the next group assignment was to that respective group as a replacement. There were no serious adverse events in either group, though the seven who did not complete RENEW dropped out due to illnesses not thought to be associated with participating in the study (n = 5) or scheduling conflicts (n = 2).

The 21 participants in the RENEW group completed an average of 34 out of the potential 36 sessions over 12 weeks of RENEW, resulting in a compliance rate of 95%. The total average RENEW per week increased over the 12 weeks of training. Following the first 2 weeks of acclimatization and ramping up of RENEW in a progressive fashion, participants increased their workload ~3-fold from week 3 (7.6 ± 5.1 kJ) to week 12 (22.1 ± 14.8 kJ).

### Measures

#### Muscle Size: Quadriceps Lean Tissue

The pre-intervention and post-intervention group mean values and within-group mean change scores (and 95% confidence intervals) for muscle size, are depicted in table 4. Significant (p < 0.05) pre-intervention differences existed between groups for quadriceps lean tissue average cross sectional area (RENEW: 43.2 ± 10.8 cm^2^; Usual-Care: 50.9 ± 11.4 cm^2^). The post-intervention quadriceps lean tissue average cross sectional area (RENEW: 44.9 ± 10.9 cm^2^; Usual-Care: 50.8 ± 11.7 cm^2^) changes resulted in a statistically significant (p = 0.001) group x time interaction effect for muscle size. The magnitude of change in the RENEW group (4% increase, ES = .16) was greater than the Usual-Care group's pre to post intervention difference. (<1% increase, ES = .01).

**Table 4:** Group (RENEW and Usual Care) pre and post intervention mean values, within group mean change scores (95% CI) and effect size, and the ANCOVA group by time interaction F-score and p-value for the muscle size, muscle strength, muscle power and mobility outcomes

	RENEW				Usual Care		
	Pre	Post	Change (95% CI) and Effect Size (ES)	Interaction Group x Time F Score (p value)	Pre Mean	Post Mean	Change (95% CI) and Effect Size (ES)
**Quadriceps Lean Tissue Average Cross Sectional Area **(cm^2^)	43.2	44.9	1.7 (1.1 to 2.3) ES = 0.16	**14.5 (0.001)†**	50.9	50.8	-0.1 (-0.8 to 0.6) ES = 0.01
**Knee Extension Peak Strength **(N)	248.3	275.4	27.1 (4.5 to 49.7) ES = 0.28	1.8 (0.19)	268.0	271	3.0 (-22.3 to 28.3) ES = 0.04
**Leg Extension Muscle Power **(W)	198.2	255.5	57.3 (24.4 to 90.2) ES = 0.71	3.6 (0.07)	239.3	258.4	19.1 (0.8 to 37.6) ES = 0.22
**Six Minute Walk **(m)	417.2	466.9	49.7 (26.0 to 73.4) ES = 0.39	**5.0 (0.03)†**	442.7	453.1	10.4 (-15.9 to 36.7) ES = 0.09
**Stair Descent **(s)	6.8	5.4	1.4 (0.5 to 2.4) ES = 0.40	**4.7 (0.04)†**	5.8	5.5	0.3 (-0.2 to 0.8) ES = 0.14

† = Significant (p < 0.05) group by time interaction via an ANCOVA.

#### Muscle Strength: Maximal Voluntary Isometric Knee Extension Peak Force

The pre-intervention and post-intervention group mean values and within-group mean change scores (and 95% confidence intervals) for muscle strength, are depicted in table 4. There were no significant (p > 0.05) pre-intervention maximal voluntary knee extension peak force differences between groups (RENEW: 248.3 ± 92.2 N; Usual-Care: 268.0 ± 73.6 N). The post-intervention maximal voluntary knee extension peak force (RENEW: 275.4 ± 99.3 N; Usual-Care: 271.0 ± 75.0 N) changes did not result in a statistically significant (p = 0.15) group x time interaction effect for muscle strength. However, the magnitude of change in the RENEW group, (11% increase, ES = .28) exceeded the Usual-Care group's pre- to post-intervention (1% increase, ES = .04).

#### Muscle Power: Stair Climbing Leg Power

The pre-intervention and post-intervention group mean values and within-group mean change scores (and 95% confidence intervals) for muscle power, are depicted in table 4. There were no significant (p > 0.05) pre-intervention stair climbing leg power differences between groups (RENEW: 198.2 ± 74.7 W; Usual-Care: 239.6 ± 82.2 W). The post-intervention stair climbing leg power (RENEW: 255.5 ± 87.3 W; Usual-Care: 258.4 ± 94.4 W) changes resulted in a statistically significant (p = 0.04) group x time interaction effect for muscle power. The magnitude of change in the RENEW group (29% increase, ES = .71) was greater than the Usual-Care group's pre- to post intervention difference (8% increase, ES = .21).

#### Mobility: Six-Minute Walk and Stair Descent Tests

The pre-intervention and post-intervention group mean values and within-group mean change scores (and 95% confidence intervals) for mobility, are depicted in table 4. There were no significant (p > 0.05) pre-intervention six-minute walk and stair descent test differences between groups (RENEW: 417.2 ± 127.1 m; Usual-Care: 442.7 ± 101.9 m; and RENEW: 6.8 ± 4.5 s; Usual-Care: 5.8 ± 2.4 s). The post-intervention six-minute walk distance (RENEW: 466.9 ± 125.1 m; Usual-Care: 453.1 ± 121.9 m) changes resulted in a statistically significant (p = 0.03) group x time interaction effect for mobility. The magnitude of the RENEW group change (12% increase, ES = .39) was greater than the Usual-Care group's pre- to post intervention difference (2% increase, ES = .09).

The post-intervention stair descent time (RENEW: 5.4 ± 2.5 s; Usual-Care: 5.5 ± 1.9 s) changes did not result in a statistically significant (p = 0.07) group x time interaction effect for mobility. However, the magnitude of the RENEW group's change (21%, ES = .40) exceeded the Usual-Care group's pre to post intervention difference (5%, ES = .14).

## Discussion

The overarching finding from this exploratory pilot study is that an older, heterogeneous group of cancer survivors can improve their muscle and mobility status with a unique form of resistance training; and that compliance is excellent with 12 weeks of the RENEW intervention. Collectively these outcomes have a high clinical impact considering the priority placed on mitigating muscle and mobility deficits in older cancer survivors [25] and that previous resistance exercise studies with older (>59 years of age) individuals have been limited to survivors of prostate cancer [11,12,26].

Cancer and its treatment typically induces muscle atrophy due to perturbations in muscle protein metabolism, including decreased muscle protein synthesis, increased muscle protein degradation, or a combination of both [27]. Therefore, the modest, but resultant increase (4%) in quadriceps lean tissue cross sectional area following RENEW is promising especially because previous resistance exercise trails with cancer survivors [8] have not demonstrated evidence of an effect on leg lean tissue cross sectional area in older cancer survivors, and a similar effect of RENEW has been noted with older prostate cancer survivors [14]. The increase (11%) in knee extension peak strength following RENEW is also favorable particularly when coupled with the large effect and increase (29%) in leg extension muscle power. Previous resistance exercise trials in cancer survivors reveal overall upper and lower body strength increases following resistance training, though very few studies [14] testing knee extension strength have demonstrated a positive effect [8]. Segal demonstrated a greater increase in lower body strength in survivors of prostate cancer following 12 [11] and 24 [12] weeks of supervised resistance training, though the participants were younger than our cohort and composed of men-only. Likewise, prostate cancer survivors from another study [26] improved their leg press strength over 90% after 20 weeks of resistance exercise, though this may be an overestimate as the mode of testing was identical to the resistance exercise mode. Finally, the large effect of RENEW on improving leg muscle power, a heretofore unreported outcome in older cancer survivors, may be the most significant muscle outcome since muscle power production is more closely related to mobility in older individuals than either muscle size or strength [28].

The improved mobility status following RENEW is a critical outcome as physical function is characterized in large part by the ability to move about, and mobility impairments are targeted by rehabilitation interventions in needy older populations like cancer survivors. After 12 weeks of RENEW a 12% increase in walking endurance resulted, which exceeds the 7% increase reported by Galvao [26] after 20 weeks of resistance exercise in older prostate cancer survivors. Notably, we also observed a 21% improvement in the ability to descend stairs safely following RENEW. This is a critical task since a preponderance of falls occur while negotiating stairs, especially during stair descent [29]. The improved mobility status in older cancer survivors following RENEW is commensurate with previously reported improvements in mobility in other older populations [14-17].

The first exercise study that targeted older cancer survivors by Denmark-Wahnefried et al [30] reported that when public health guidelines for vigorous exercise were met higher scores on physical functioning resulted. Unfortunately, adherence to exercise is the lowest (<65%) for the oldest cancer survivors enrolled in clinical trials [31]. Because RENEW requires only "somewhat hard" levels of perceived exertion to induce positive muscle and mobility responses in 70+ year old cancer survivors, it may be a reason for its excellent compliance rate (95%) which exceeds that of younger (<65 years of age) cancer survivors [32]. The most recent exercise study that targeted older cancer survivors by Morey et al [33] included a heterogeneous cohort that was nearly identical to ours and consisted of 70+ year old males and females who were 8-9 year survivors of breast, prostate and colorectal cancers. Those who exercised in this study experienced a less rapid deterioration of physical function over a one-year period after following a home-based diet and exercise regimen. In our study a supervised RENEW program followed for less than one-fourth of the time (12 weeks) reversed a functional decline rather than simply ameliorated a functional decline. This reversal of deficits in physical function has been documented following RENEW in other older populations [14-17].

This study of RENEW with older cancer survivors fills a void in the resistance exercise literature as a lack of information regarding the optimal type, intensity, and duration of physical exercise exists [34]. There are, however, limitations to this exploratory trail therefore the results should be interpreted with caution. This study was exploratory in nature and utilized a restricted randomization approach to achieve balance in size between the groups and to have adequate experience with the intervention and enough participants for comparison. A future randomized clinical trial is needed to substantiate the findings. Further, the heterogeneous cohort of males and females who have survived various cancer types and treatments prevents a generalization of the results to specific subgroups of cancer survivors. This heterogeneity, however, represents the diversity of older individuals who have survived cancer and who are limited in both their muscle and mobility status and hence are in need of rehabilitation countermeasures. Likewise, this exploratory trial's lack of control of attention, physical activity levels and general health related behaviors during the intervention period may confound the results. The RENEW intervention's effect, however, was robust enough to withstand inherent variability amongst the cohorts and lack of control of potentially confounding variables. Overall, we believe the results represent a conservative estimate of the potential efficacy of RENEW for older cancer survivors, due to the relatively small sample size, a larger trial with an intent to treat approach is needed to determine RENEW's overall effectiveness.

## Conclusions

This exploration of RENEW in a heterogeneous cohort of older cancer survivors demonstrates increases in muscle size, strength and power along with improved mobility.

## Competing interests

One of the authors (PCL) is a co-inventor on the ergometer licensed to Eccentron; BTE Technologies, Inc., Hanover, MD, USA. Neither PCL nor any of the other authors, have received any financial incentives (e.g., reimbursements, fees, royalties, funding, or salary) from the company or stemming from the contents of this manuscript or any related published papers.

## Authors' contributions

All authors made substantial contributions to conception and design, or acquisition of data, or analysis and interpretation of data; were involved in drafting the manuscript; and have given final approval of the version to be published. Specifically, PCL conceived of the study; PCL, RLM, LED and SLB participated in the design of the study; SBS provided the exercise training and oversaw data collection; and LED performed the statistical analysis.

## Pre-publication history

The pre-publication history for this paper can be accessed here:

http://www.biomedcentral.com/1471-2318/11/5/prepub
